# Plasma extracellular vesicles in meningioma patients following radiotherapy as liquid biopsy- a prospective explorative biomarker study (ARO 2023-05/AG-NRO-07)

**DOI:** 10.1186/s12885-024-12170-4

**Published:** 2024-04-11

**Authors:** Maximilian Y. Deng, Amanda Salviano da Silva, Pauline Carlotta Göller, Laila König, Henning Schäfer, Cecile Maire, Adriane Lentz-Hommertgen, Thomas Held, Sebastian Regnery, Tanja Eichkorn, Florian Stritzke, Lukas Bauer, Daniel Schnell, Klaus Herfarth, Andreas von Deimling, Sandro Krieg, Antje Wick, Wolfgang Wick, Anca Grosu, Jürgen Debus, Felix Sahm, Franz Ricklefs

**Affiliations:** 1 Department of Radiation Oncology, Heidelberg University Hospital, Heidelberg University, Heidelberg, Germany; 2 Heidelberg Institute for Radiation Oncology (HIRO) and National Center for Radiation Research in Oncology (NCRO), Heidelberg, Germany; 3 National Center for Tumor Diseases (NCT), NCT Heidelberg, a partnership between DKFZ and Heidelberg University Hospital, Heidelberg, Germany; 4 Heidelberg Ion-Beam Therapy Center (HIT), Department of Radiation Oncology, Heidelberg University Hospital, Heidelberg University, Heidelberg, Germany; 5 Department of Neurosurgery, University Medical Center Hamburg-Eppendorf, Hamburg, Germany; 6 Department of Neuropathology, CCU Neuropathology, Heidelberg University Hospital, Heidelberg University, German Consortium for Translational Cancer Research (DKTK), German Cancer Research Center (DKFZ), Heidelberg, Germany; 7 Department of Radiation Oncology, University Medical Center Freiburg, University of Freiburg, Freiburg, Germany; 8 Department of Neurosurgery, University Hospital Heidelberg, Heidelberg University, Heidelberg, Germany; 9 Department of Neurology, Heidelberg University Hospital, Heidelberg University, Heidelberg, Germany; 10 Clinical Cooperation Unit Neurooncology, German Consortium for Translational Cancer Research (DKTK), German Cancer Research Center (DKFZ), Heidelberg, Germany; 11 Clinical Cooperation Unit Radiation Oncology, German Consortium for Translational Cancer Research (DKTK), German Cancer Research Center (DKFZ) Heidelberg, Heidelberg, Germany

**Keywords:** Radiotherapy, Liquid biopsy, Extracellular vesicles, Meningioma.

## Abstract

**Background:**

While surgical resection remains the primary treatment approach for symptomatic or growing meningiomas, radiotherapy represents an auspicious alternative in patients with meningiomas not safely amenable to surgery. Biopsies are often omitted in light of potential postoperative neurological deficits, resulting in a lack of histological grading and (molecular) risk stratification. In this prospective explorative biomarker study, extracellular vesicles in the bloodstream will be investigated in patients with macroscopic meningiomas to identify a biomarker for molecular risk stratification and disease monitoring.

**Methods:**

In total, 60 patients with meningiomas and an indication of radiotherapy (RT) and macroscopic tumor on the planning MRI will be enrolled. Blood samples will be obtained before the start, during, and after radiotherapy, as well as during clinical follow-up every 6 months. Extracellular vesicles will be isolated from the blood samples, quantified and correlated with the clinical treatment response or progression. Further, nanopore sequencing-based DNA methylation profiles of plasma EV-DNA will be generated for methylation-based meningioma classification.

**Discussion:**

This study will explore the dynamic of plasma EVs in meningioma patients under/after radiotherapy, with the objective of identifying potential biomarkers of (early) tumor progression. DNA methylation profiling of plasma EVs in meningioma patients may enable molecular risk stratification, facilitating a molecularly-guided target volume delineation and adjusted dose prescription during RT treatment planning.

## Background

Meningiomas comprise a family of neoplasms derived from meningothelial cells of the arachnoid mater, representing the most common primary intracranial tumor in adults [1]. Conventional histology-based WHO classification allotted 3 different grades to meningiomas for risk stratification - with increasing malignancy and risk of recurrence, ranging from WHO grade 1 to 3 ^1^. Meningioma cells were reported to secrete extracellular vesicles (EVs) (e.g. exosomes, microvesicles and oncosomes) into the bloodstream, which can be isolated for liquid biopsy [2–4]. A recent study by Ricklefs et al. investigated the dynamic of plasma EVs in meningioma patients following surgical resection. At initial diagnosis, meningioma patients displayed elevated levels of circulating EVs, as compared to healthy individuals. While preoperative plasma EV counts were positively correlated with WHO grading, decline of plasma EVs was reported to be associated with the extent of resection following surgical resection. Notably, plasma EV levels were not directly associated with tumor size, but correlated with the extent of tumor edema, indicating that leakiness of the blood-brain barrier may be responsible for the elevated levels of circulating EVs. Subsequent molecular classification revealed that DNA methylation profiling of EV-DNA may allow accurate allocation of patients into methylation subclasses, facilitating molecular risk stratification [2].

While surgical resection remains the primary treatment approach for symptomatic or growing meningiomas, fractionated external beam radiotherapy (RT) represents an auspicious alternative treatment approach in patients with meningiomas not safely amenable to surgery. Here, biopsies are often omitted in light of potential iatrogenic neurological deficits, resulting in an absence of histological grading and (molecular) risk stratification [1, 5, 6].

The present study aims to investigate the dynamic of plasma EVs in meningioma patients during radiotherapy and subsequent follow-up, which may serve as biomarker in treatment response and disease monitoring (e.g. early detection of relapse). Further, DNA methylation profiling of enriched plasma EVs may facilitate molecular risk stratification in meningioma patients, allowing a molecularly-guided target volume delineation and adjusted dose prescription during RT treatment planning.

## Methods/design

**Fig. 1:**
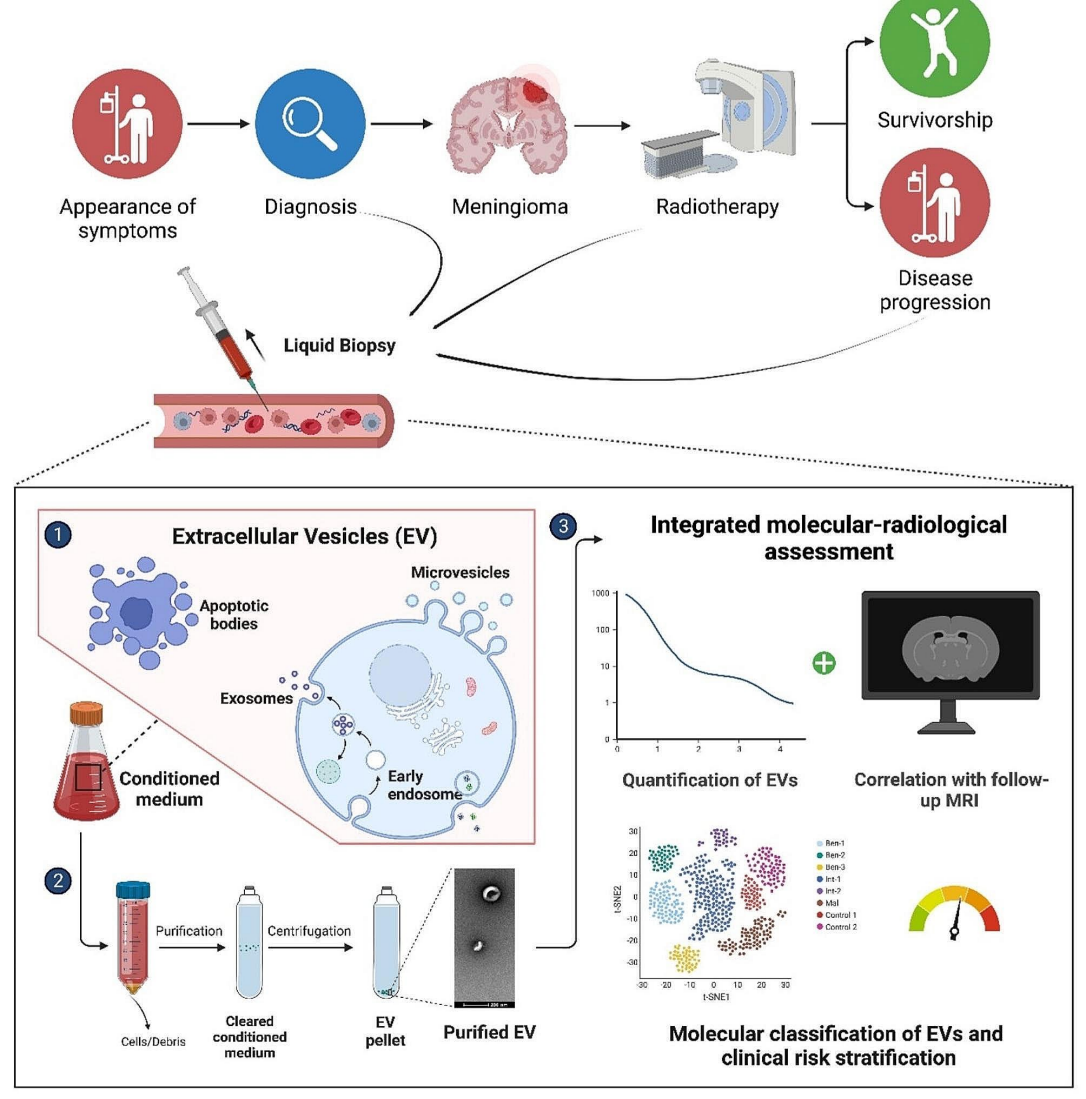
Study illustration

The study will be performed as a prospective, multicenter, explorative biomarker study. A total of 60 patients are projected to be enrolled into the study. Patients fulfilling the inclusion criteria will be treated with either photon- (intensity-modulated or 3D-conformal) or particle radiotherapy. The overall duration of the trial is scheduled to be 36 months, consisting of 24 months of recruitment and a minimum follow-up of 6 months. A flow chart for trial subjects is shown in Fig. 1.

### Inclusion criteria

Inclusion criteria are defined as: macroscopic tumor in MRI (either as definitive RT, or following subtotal resection or relapse), confirmed meningioma (histologically or MRI/DOTATOC-PET CT), indication for radiotherapy, completed wound healing after surgical intervention, Karnofsky Performance Score ≥ 60%; age ≥ 18 years, written informed consent, ability of subject to understand character and individual consequences of the trial, and adequate contraception for women of childbearing potential.

### Exclusion criteria

Exclusion criteria are defined as: previous or known tumor diseases < 5 years ago, previous (cerebral) radiotherapy, simultaneous chemo/immunotherapy, evidence that the patient cannot adhere to the study protocol (e.g., non-compliance), the refusal of patients to participate in the study and participation in another clinical study or observation period in a competing trial.

### Radiation therapy

Radiation treatment will be carried out according to the current standard of care, following current clinical guidelines for meningiomas [6]. Target volume definition, the selection of treatment modality, total dose and fractionation are not study-specific. Simultaneous systemic therapies, particularly chemo- or immunotherapy, are not allowed in the current study. Patients will be immobilized with an individual immobilization mask for treatment planning.

All patients will receive a non-contrast planning CT scan (with a layer thickness of 3 mm) and a magnetic resonance imaging (MRI) for an optimal definition of the target volume. Dose constraints for normal tissues and organs will be respected according to the Quantitative Analyses of Normal Tissue Effects in the Clinic (QUANTEC) [7, 8]. According to the standard of care, intensity-modulated (photon) radiotherapy (IMRT) will be performed 5 fractions per week under image guidance with daily CT images and position correction using volumetric modulated arc therapy. If indicated, particle therapy will be carried out 5–6 fractions per week with protons or carbon ions, using active raster scanning for orthogonal X-ray image guidance and daily position correction. The target volume and the dose prescription will be defined at discretion of the treating radiation oncologist, following current clinical guidelines for meningiomas [6].

### Study visits

Clinical follow-up will be performed to clinical guidelines [6] and will not be study-specific, with exception of the collection of blood samples (EDTA). Patients will be followed-up for at least 6 months after radiotherapy, and thereafter every 6 months. Treatment response and progression will be defined according to the current guidelines established by the RANO working group [9].

**Fig. 2:**
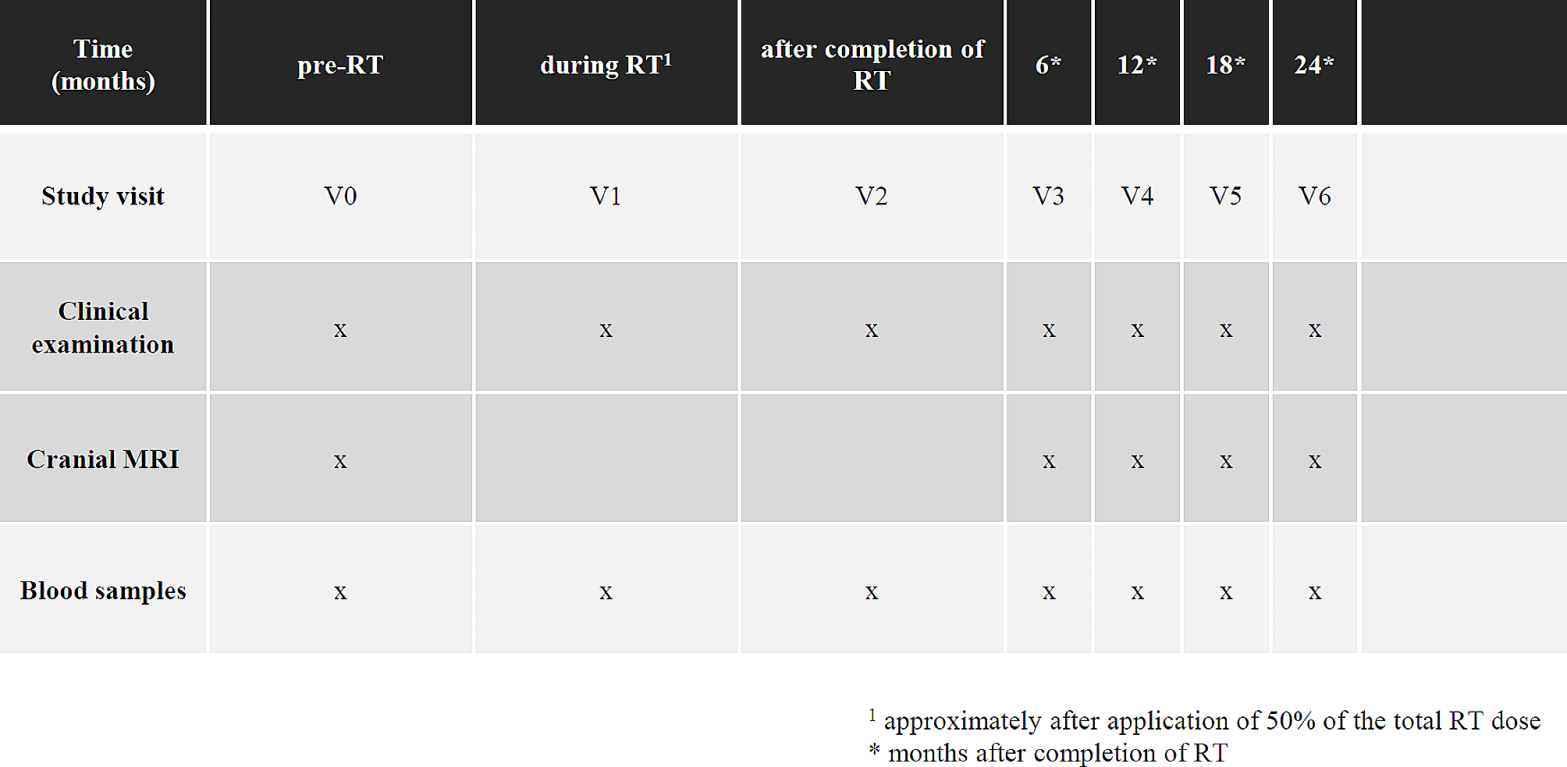
Study visits

Blood samples (EDTA) will be collected before start of radiotherapy, after application of approx. 50% of the total dose, after completion of radiotherapy and during clinical follow-up every 3–6 months until 2 years following radiotherapy. At each timepoint, 3 EDTA blood tubes à 7 ml will be withdrawn. The study visit plan is shown in Fig. 2.

### Study endpoints

The primary aim of this study is to analyze the reduction of plasma EVs in meningioma patients following radiotherapy, as compared to the baseline prior to radiotherapy. This study is planned as an exploratory trial and therefore no formal sample size calculation was performed. Based on Ricklefs et al. [2], we assume a mean of 1.643 for the change in EVs with a standard deviation of 3.750. With 48 patients having values after 2 years (60 patients included with 20% drop-outs assumed), this results in a 95% confidence interval of width 2.178 [0.554; 2.732]. Descriptive statistics regarding changes at different time points including calculation of 95% confidence intervals. Changes over time will be graphically visualized. All patients with data available will be considered.

Secondary endpoints will be evaluated in all patients. Descriptive analysis (including Kaplan-Meier estimates for PFS and OS) will be provided using adequate statistics and calculation of 95% confidence intervals. Reasons for missing values will be described and considered in the interpretation of results. During clinical follow-up, the quantity of plasma EVs will be correlated with treatment response or tumor progression on MRI according to RANO guidelines, to address a potential correlation between level of plasma EVs and (early) tumor progression [9]. Further, methylation profiles of plasma EV-DNA will be generated for further molecular risk stratification into existing methylation families in meningioma [5]. If available (e.g. following resection or biopsy), further comparison, with the methylation family of the meningioma will be performed to validate the methylation classification of plasma EV-DNA.

### Blood sample processing, isolation and imaging flow cytometry (IFCM) of extracellular vesicles

EVs will be isolated from plasma by differential ultracentrifugation and analyzed using nanoparticle tracking analysis (NTA), as described previously [2]. In brief, plasma supernatants will be centrifuged to eliminate cell debris, filtered, and subsequently washed with PBS. The concentration and size of EVs will be assessed by nanoparticle tracking analysis (NTA) via LM14 instrument (NanoSight, Malvern Panalytical) equipped with a 638 nm laser and a Merlin F-033B IRF camera (Adept Electronic Solutions). Antibodies, including anti-CD9 (Biolegend, clone HI9a, PE), anti-CD81 (Biolegend, clone 5A6) and anti-CD63 (Biolegend, clone H5C6, PacificBlue) will be used to stain EV-specific tetraspinins for imaging flow cytometry (IFCM) analysis to confirm the presence and to further characterize the expression of EV-surface proteins. EV data will be acquired on an AMNIS ImageStreamX Mark II Flow Cytometer (AMNIS/Millipore, Seattle), as previously described [10].

### DNA methylation profiling of plasma EVs using nanopore-sequencing

DNA will be isolated from plasma EVs using the XCF exosomal DNA isolation kit and quantified by Qubit (Invitrogen). Extracted EV-DNA will be sheared to 9–11 kb in a total volume of 50 µl using g-TUBEs (Covaris) at 7200 rpm for 120 s. Fragment length will be assessed using Agilent 2100 Bioanalyzer (Agilent Technologies).

Sequencing libraries will be prepared with the SQK-LSK109 Ligation Sequencing Kit, as previously described [11]. In brief, 48 µl of sheared DNA (2–2.5 µg) will be incubated for 30 min at 20 °C, then for 30 min at 65 °C followed by a cool down to 4 °C. Subsequent clean-up will be performed using AMPure XP beads and 80% ethanol with an elution time of 5 min, followed by adapter ligation for 60 min at room temperature. The ligation mix will be incubated with AMPure XP beads at 0.4x for 10 min and cleaned-up using the Long Fragment Buffer. The final library will be eluted in a total volume of 31 µl, as previously described [11]. Library concentrations will be assessed by the Invitrogen Qubit DNA HS Assay Kit (Thermo Fisher Scientific) using a benchtop Quantus fluorometer (Promega). The libraries will be loaded (500–600 ng) onto FLO-MIN106 R9.4.1 flow cells with a minimum of 1100 pores available according to the FC Check prior to loading. Flow cells will be flushed after around 24 h for a total of two times per sample with the Flow Cell Wash Kit, according to the manufacturer’s instructions. Sequencing will be performed using a MinION 1B and GridION (Oxford Nanopore Technologies).

Methylation calls are made using megalodon v2.3.3 with a guppy v5.0.11 backend, as previously described [11]. Classification of nanopore sequencing derived DNA-methylation profiles was established using a random forest classifier based on nanoDx, which was previously trained on the publicly available 450k/EPIC methylation array reference data set of the Heidelberg methylation classifier version v12.8 ^11–13^. In brief, for each nanopore sample, methylation calls overlapping the top 100,000 probes derived from the Heidelberg methylation classifier were selected. Subsequently, these probes were variance filtered to select the top 10,000 most variable probes, and a random forest model with 20,000 trees was trained, as previously described [11]. A prediction and confidence score for the sample was generated. Methylation families were determined by aggregating over methylation subclasses from the reference set [11, 12].

### Ethics and safety considerations

Data collection, management and processing will be performed using the in-house research database. The study protocol, patient information sheet, and declaration of informed consent were approved by the Heidelberg University Ethics Committee (S-277/2023). The study will be conducted in accordance with Good Clinical Practice guidelines and the Declaration of Helsinki. The regulations concerning medical confidentiality and data protection are fulfilled. Adverse events will be monitored and documented according to GCP guidelines. The study is registered in ClinicalTrials.gov (Identifier: NCT06104930, registration date: October 27th, 2023).

## Discussion

The primary purpose of this prospective, explorative biomarker study is to evaluate whether the level of plasma EVs can serve as biomarker in disease monitoring following radiotherapy. A recent landmark study by Ricklefs et al. has reported an elevated level of plasma EVs in meningioma patients, as compared to healthy individuals. Notably, a decrease in plasma EV counts was encountered after surgical resection. Further, plasma EV levels were associated with the extent of tumor edema, thus, Ricklefs et al. discussed the potential role of the leakiness of the blood-brain barrier on the elevated levels of circulating EVs [2]. To date, the dynamic of EVs during and after radiotherapy remains enigmatic. Previous reports have indicated that a temporary contrast enhancement and, by association a transient disruption of the blood-brain barrier, may be observed in a subset of patients following cranial RT [13, 14]. A radiation-induced “washout” of meningioma EVs into the bloodstream may be encountered, potentially providing sufficient plasma EV counts for further molecular classification.

In summary, this study will investigate the level of plasma EVs and its dynamic under/after radiotherapy, facilitating our understanding of the effect of radiotherapy on EV biology. Further, treatment response and progression will be correlated with the level of plasma EVs, ultimately with the hope of identifying potential biomarkers of (early) tumor progression. Further, DNA methylation profiling of plasma EVs in meningioma patients may provide a molecular risk stratification, leading to a molecularly-guided target volume delineation and adjusted dose prescription during RT treatment planning.

## Data Availability

No datasets were generated or analysed during the current study.

## Abbreviations

CT: Computer tomography
CTV: Clinical target volume
EDTA: Ethylenediaminetetraacetic acid
EV: Extracellular vesicles
GCP: Good Clinical Practice
MRI: Magnet resonance imaging
NTA: Nanoparticle tracking analysis
RANO: Response Assessment in Neuro-Oncology
RT: Radiation therapy
WHO: World Health Organisation
